# The State Cancer Laboratory

**Published:** 1911-03

**Authors:** 


### The State Cancer Laboratory

THERE is every prospect that the bill introduced by state Senator Loomis, carrying an appropriation of $65,000 for the erection of a hospital for the purpose of cancer research work in connection with the State Cancer Laboratory at Buffalo, will pass the legislature of the state of New York and become a law by signature of Governor Dix.

This is the measure referred to in the last issue of the BUFFALO MEDICAL JOURNAL and commended as being of the utmost importance. While every sign of political import points to a successful voyage for the measure through the shoals of legislative passage, it should not be inferred that these pointings do away with the necessity for watchfulness and serious effort to bring about that easement of passage which the humanitarian elements of the bill and its supreme importance to scientific advancement deserve. Every physician should bring himself into close touch with his assemblyman and senator and urge their efforts for the bill irrespective of party politics. A campaign of personal appeal and direct correspondence should be instituted and the legislators pledged to the measure; not only by individual support but by open advocacy.

The work which has been done and that under way at the State Cancer Laboratory is of inestimable value. The investigations which have been carried to a successful issue and those which give every promise of producing results in the near future which will have the effect of revolutionising treatment of malignant disease are of such an epoch-making nature as to warrant the expenditure by the state of New York of a much larger sum of money than is asked for in the bill under consideration. The amount is really small, and were it tenfold, there would be sufficient reason for careful consideration before it were incontinently refused on any ground of false economy.

The recent newspaper agitation in connection with the work done at the laboratory is regrettable. The fact that newspapers have been placed in position to demand, on the ground of public policy, proofs of the alleged official statements of the absolute cure of malignant disease is the natural outcome of appeal to legislative support. The JOURNAL has always maintained that the best way to educate the public in scientific matters is to take the public into the confidence of the scientists and preferably through the columns of the daily newspapers. The lay reader is thus reached direct and statements of fact are placed before him in common sense fashion and without any of that veneer of mystery which used to be the cloak of the ancient physician who dabbled in drugs and dogmatics and shed signs and symbols in his treatment of disease.

The laboratory workers are doing a service to humanity which is beyond the limits of the lay mind. They are paving the way for the mastery of one of the most horrible of the afflictions of the race; they are devoting days and nights to delving into the depths of disease, searching ceaselessly for its definite cause and seeking that which will at least arrest if it does not absolutely cure. The JOURNAL believes there will come out of this work, some day, some time in the no distant future, a cure for malignant disease and this statement is made on definite knowledge of what is and not merely what may be.

The medical profession should without delay communicate with the members of the state legislature and urge the passage of the bill. This should not be done by mere printed petition; it should be a personal campaign promptly begun and persistently continued until the Governor affixes his signature to the measure, which gives to the State Cancer Laboratory additional facilities for the pursuit of ultimate discovery of one of the greatest boons which can be conferred on humanity.

